# Risk factors for secondary hemophagocytic lymphohistiocytosis in severe coronavirus disease 2019 adult patients

**DOI:** 10.1186/s12879-021-06094-8

**Published:** 2021-04-29

**Authors:** Mei Meng, Limin Chen, Sheng Zhang, Xuan Dong, Wenzhe Li, Ranran Li, Yunxin Deng, Tao Wang, Yan Xu, Jiao Liu, Yanxia Huang, Yizhu Chen, Sisi Huang, Zhenliang Wen, Lidi Zhang, Hangxiang Du, Yongan Liu, Djillali Annane, Jieming Qu, Dechang Chen

**Affiliations:** 1 Department of Critical Care Medicine, Ruijin Hospital, Shanghai Jiao Tong University School of Medicine, No 197, Rui Jin 2nd road, Shanghai, 200025 China; 2 Department of Critical Care Medicine, Ruijin Hospital North, Shanghai Jiao Tong University School of Medicine, Shanghai, China; 3 Tuberculosis and Respiratory Department, Wuhan Infectious Diseases Hospital, Wuhan, China; 4 General intensive care unit, Raymond Poincaré Hospital (APHP), Laboratory of Inflammation and Infection U1173, University of Versailles SQY/INSERM 104 bd Raymond Poincaré, 92380 Garches, France; 5 Department of Pulmonary and Critical Care Medicine, Ruijin Hospital, Shanghai Jiao Tong University School of Medicine, Shanghai, China; 6 Institute of Respiratory Diseases, Shanghai Jiao Tong University School of Medicine, Rui Jin 2nd road, Shanghai, 200025 China

**Keywords:** Secondary Hemophagocytic Lymphohistiocytosis, COVID-19, HScore, Ferritin, Triglycerides, Platelet, Cytokine storm

## Abstract

**Background:**

Secondary hemophagocytic lymphohistiocytosis (sHLH) is a life-threatening hyperinflammatory event and a fatal complication of viral infections. Whether sHLH may also be observed in patients with a cytokine storm induced by severe acute respiratory syndrome coronavirus 2 (SARS-CoV-2) infection is still uncertain. We aimed to determine the incidence of sHLH in severe COVID-19 patients and evaluate the underlying risk factors.

**Method:**

Four hundred fifteen severe COVID-19 adult patients were retrospectively assessed for hemophagocytosis score (HScore). A subset of 7 patients were unable to be conclusively scored due to insufficient patient data.

**Results:**

In 408 patients, 41 (10.04%) had an HScore ≥169 and were characterized as “suspected sHLH positive”. Compared with patients below a HScore threshold of 98, the suspected sHLH positive group had higher D-dimer, total bilirubin, alanine aminotransferase, aspartate aminotransferase, blood urea nitrogen, serum creatinine, triglycerides, ferritin, interleukin-6, C-reactive protein, procalcitonin, lactate dehydrogenase, creatine kinase isoenzyme, troponin, Sequential Organ Failure Assessment (SOFA) score, while leukocyte, hemoglobin, platelets, lymphocyte, fibrinogen, pre-albumin, albumin levels were significantly lower (all *P* < 0.05). Multivariable logistic regression revealed that high ferritin (>1922.58 ng/mL), low platelets (<101 × 10^9^/L) and high triglycerides (>2.28 mmol/L) were independent risk factors for suspected sHLH in COVID-19 patients. Importantly, COVID-19 patients that were suspected sHLH positive had significantly more multi-organ failure. Additionally, a high HScore (>98) was an independent predictor for mortality in COVID-19.

**Conclusions:**

HScore should be measured as a prognostic biomarker in COVID-19 patients. In particular, it is important that HScore is assessed in patients with high ferritin, triglycerides and low platelets to improve the detection of suspected sHLH.

## Highlights

- Secondary hemophagocytic lymphohistiocytosis (sHLH) has prognostic value in corona virus disease 2019 (COVID-19)
- HScore in severe COVID-19 cases can help detect sHLH
- Risk factors for suspected sHLH in severe COVID-19 are high ferritin (>1922.58 ng/mL), low platelets (<101 × 10^9^/L) and high triglycerides (>2.28 mmol/L).
- Cytokine storm syndrome is the cause of suspected sHLH in severe COVID-19

## Introduction

COVID-19 caused by SARS-CoV-2 continues to spread globally and represents a serious worldwide health problem [1]. RECOVERY trial results showed dexamethasone reduced 28-day mortality in severe COVID-19 by attenuating the exaggerated inflammatory response in the host [2]. However, in coronavirus infection, several studies observed delay in viral clearance with systemic corticosteroid therapy, potentially indicating increased viral replication as an adverse effect [1]. Therefore, it is urgent to accurately identify the patient subsets that may benefit from immunosuppressive treatment.

Accumulating evidence has indicated that a cytokine storm caused by an excessive immune response drives disease progression and organ failure in COVID-19 patients [3–5]. sHLH is a cytokine-driven fulminant hyperinflammatory syndrome associated with morbidity and mortality up to 88%. sHLH in adults often occurs after infections, especially viral infections by Epstein-Barr Virus (EBV), cytomegalovirus (CMV), human immunodeficiency virus (HIV), and influenza [6]. The main clinical features of sHLH include persistent fever, cytopenia, and hyperferritinemia [7]. Lung involvement is common and was shown to be an independent determinant of death in sHLH [8]. It is reasonable to speculate that the abnormal hyperinflammatory state in COVID-19 may induce sHLH, thus driving a poor prognosis.

A recent study suggested that HScore, a score for the diagnosis of reactive hemophagocytic syndrome, could be used to identify suspected occurrence of sHLH in severe COVID-19 without mandatory bone marrow hemophagocytosis [3]. Once a diagnosis of sHLH is established, targeted interventions should be carried out rapidly, including immunosuppressants such as glucocorticoids and chemotherapy drugs [7]. To provide more accurate diagnosis and appropriate treatment, it is necessary to conduct studies about sHLH in severe COVID-19.

In this study, we screened severe COVID-19 patients for sHLH with objectives to (1) determine the incidence of suspected sHLH in severe COVID-19, (2) describe clinical features of suspected sHLH associated with COVID-19, (3) evaluate risk factors for suspected sHLH and identify mortality-associated risk factors in COVID-19 patients, and (4) highlight potential relationships between emerging knowledge of COVID-19 disease progression and suspected sHLH.

## Patients and methods

The present study is a single-center, retrospective study, which was approved by the ethics committee of Wuhan Infectious Diseases Hospital (KY-2020-03-01).

### Data collection

A total of 1399 confirmed COVID-19 patients from January 2, 2020 to March 28, 2020 were screened. We retrieved demographic and epidemiological characteristics, laboratory testing results, clinical diagnosis and treatment data from electronic medical records and files.

### Definition

Severe COVID-19 was defined according to WHO 2020 classification, as follows: fever or suspected respiratory infection, plus one of the following: respiratory rate > 30 breaths/min; severe respiratory distress; or SpO_2_ ≤ 93% on room air [9]. The Berlin Definition was used to diagnose acute respiratory distress syndrome (ARDS) [10]. Acute kidney injury (AKI) was diagnosed using the KDIGO criteria [11]. Prothrombin time test, the levels of fibrin/fibrinogen (Fib) degradation products, and platelet counts were used for the diagnosis of disseminated intravascular coagulation (DIC) [12]. Diagnosis of septic shock was based on the Third International Consensus Definition for Sepsis and Septic Shock [13]. Liver and heart injury were diagnosed according to laboratory testing results [14].

The HScore was developed by Fardet et al. and colleagues to estimate an individual’s risk of having sHLH. This scoring system can be freely available online (http://saintantoine.aphp.fr/score/) and contains nine items in routine practice, including known underlying immunodepression, maximal temperature (°C), hepatomegaly, splenomegaly, lower hemoglobin level, lower leucocytes count, lower platelets count, higher ferritin level (ng/ml), higher triglyceride level (mmol/l), lower fibrinogen level (g/l), higher serum glutamic oxaloacetic transaminase/ recombinant aspartate aminotransferase (SGOT/ASAT) level (UI/L), hemophagocytosis features on bone marrow aspirate. We set HScore 169 and 98 as the cut off because HScore at 169 achieved the optimal discriminative power in previous study and HScore < 98 virtually rules out the diagnosis of sHLH [15]. Patients with HScores between 98 and 169 were not categorized as either suspected sHLH positive or negative [15].

### Statistical analysis

Continuous variables were presented as median (lower quartile, upper quartile) and compared using the Wilcoxon rank-sum or Kruskal-Wallis tests. Frequency was used to represent the unordered classification variables and Pearson chi-square tests were used for comparison. Ordered categorical variables were expressed as frequency and compared by Wilcoxon rank-sum or Kruskal-Wallis tests. A binary logistic regression model was used for multivariate analysis to select factors associated with suspected sHLH or death. The receiver-operator characteristic analysis and area under the curve were used to evaluate the discrimination of potential variables to diagnose suspected sHLH. The model was validated internally by bootstrap resampling, and the prognostic performance was measured by the concordance index (C-index) and calibration curve. All analyses were performed using SAS software, version 9.4 (SAS Institute Inc., Cary, NC) or R software. Graphs were made in Stata 16.0 or MedCalc softwares. All probability calculations were two-tailed, and *p* values of 0.05 or less were considered to be statistically significant.

## Results

### Clinical characteristics and laboratory findings of severe COVID-19

Of the 1399 COVID-19 confirmed patients who met a treatment endpoint, either recovery or death, in Wuhan Infectious Diseases Hospital by March 28, 2020 were included in this study. Of 1399 patients, 424 met the criteria for severe COVID-19. However, 9 patients were missing, and thus 415 patients were included in this study for analysis. Among the 415 patients, 171 were female (41.20%) and 244 were male (58.80%), 255 were over than 60 years old (61.45%). Non-survivors were 220 (53.01%), including 138 male (62.73%).

Compared with survivors, non-survivors were older and had shorter length of hospital stay, higher SOFA scores and APACHE II scores at 24 h post-admission, significantly lower leukocytes, platelets, neutrophils, lymphocytes, Fib, pre-albumin, albumin, while D-Dimer, aspartate aminotransferase (AST), blood urea nitrogen (BUN), serum creatinine (sCr), ferritin, Interleukin − 6 (IL-6), C-reactive protein (CRP), procalcitonin (PCT), lactate dehydrogenase (LDH), creatine kinase isoenzyme (CK-MB), troponin were all significantly higher (all *P* < 0.0001) (Table 1). There were no significant differences in underlying comorbidities between survivor and non-survivor patients.

**Table 1:** Characteristics of all 415 patients with severe COVID-19 and stratified into survivors and non-survivors

	All(*n* = 415)	Survivors(*n* = 195)	Nonsurvivors(*n* = 220)	*P* value
Gender, Male/Female, n (%)	244(58.80) /171(41.20)	106(54.36)/ 89(45.64)	138(62.73)/ 82(37.27)	0.0839
Age, years, Mean (SD)	62.63(13.49)	57.77(12.67)	66.93(12.74)	< 0.0001
age, years, n(%)				< 0.0001
<40	23(5.54)	15(7.69)	8(3.64)	
40–60	137(33.01)	92(47.18)	45(20.45)	
≥ 60	255(61.45)	88(45.13)	167(75.91)	
ARDS, n(%)	258(62.93)/ 152(37.07)^a^;5	75(38.46) /120(61.54)	183(85.12)/ 32(14.88); 5	< 0.0001
DIC, n(%)	20(5.00) /380(95.00); 15	1(0.52) /193(99.48); 1	19(9.22) /187(90.78); 14	< 0.0001
Liver injury, n(%)	78(19.50) /322(80.50); 15	26(13.40) /168(86.60); 1	52(25.24)/ 154(74.76); 14	0.0028
Heart injury, n(%)	104(26.00) /296(74.00); 15	12(6.19) /182(93.81); 1	92(44.66)/ 114(55.34); 14	< 0.0001
Kidney injury, n(%)	60(15.00) /340(85.00); 15	5(2.58) /189(97.42); 1	55(26.70)/ 151(73.30); 14	< 0.0001
Shock, n (%)	87(21.75) /313(78.25); 15	2(1.03)/ 192(98.97); 1	85(41.26)/ 121(58.74); 14	< 0.0001
Smoking history, n(%)	20(4.84) /393(95.16); 2	15(7.69)/ 180(92.31)	5(2.29)/ 213(97.71); 2	0.0107
Alcohol history, n(%)	21(5.08)/ 392(94.92); 2	15(7.69)/ 180(92.31)	6(2.75)/ 212(97.25);2	0.0225
**Comobidities**				
Diabetes, n(%)	79(19.13) /334(80.87); 2	30(15.38)/ 165(84.62)	49(22.48) /169(77.52); 2	0.0673
Hypertension, n(%)	147(35.59)/ 266(64.41); 2	64(32.82)/ 131(67.18)	83(38.07)/ 135(61.93); 2	0.2657
Chronic heart failure, n(%)	39(9.44) /374(90.56); 2	12(6.15)/ 183(93.85)	27(12.39)/ 191(87.61); 2	0.0306
COPD, n(%)	13(3.15) /400(96.85); 2	3(1.54)/ 192(98.46)	10(4.59)/ 208(95.41); 2	0.0765
Stroke, n(%)	26(6.30)/ 387(93.70); 2	10(5.13)/ 185(94.87)	16(7.34)/ 202(92.66); 2	0.3557
Malignant Tumor, n(%)	19(4.60) /394(95.40); 2	10(5.13)/ 185(94.87)	9(4.13)/ 209(95.87); 2	0.6283
Chronic liver disease, n(%)	14(3.39)/ 399(96.61); 2	5(2.56)/ 190(97.44)	9(4.13)/ 209(95.87); 2	0.3805
Chronic kidey disease, n(%)	11(2.66) /402(97.34); 2	3(1.54)/ 192(98.46)	8(3.67)/ 210(96.33); 2	0.1793
Hepatitis B, n(%)	15(3.62) /399(96.38); 1	7(3.59)/ 188(96.41)	8(3.65)/ 211(96.35); 1	0.9726
HIV, n (%)	0(0.00) /414(100.00); 1	0(0.00)/ 195(100.00)	0(0.00)/ 219(100.00); 1	1.0000
Autoimmue disease, n(%)	17(4.12) /396(95.88); 2	7(3.59)/ 188(96.41)	10(4.59)/ 208(95.41); 2	0.6105
**Respiratory support, n(%)**				< 0.0001
Intranasal oxygen inhalation	146(37.15)	122(63.21)	24(12.00)	
Mask oxygen Inhalation	18(4.58)	10(5.18)	8(4.00)	
High Flow	67(17.05)	24(12.44)	43(21.50)	
Non-invasive ventilation	75(19.08)	9(4.66)	66(33.00)	
Invasive ventilaiton	53(13.49)	3(1.55)	50(25.00)	
ECMO	4(1.02)	0(0.00)	4(2.00)	
CRRT, n(%)	79(19.13) /334(80.87); 2	30(15.38)/ 165(84.62)	49(22.48)/ 169(77.52); 2	0.0673
Inotropic support, n(%)	87(20.96)/328(79.03)	2(1.03)/ 193(98.97)	85(38.64)/ 135(61.36)	< 0.0001
Corticosteroid treatment, n(%)	226(54.45)/189(45.54)	85(43.59)/ 110(56.41)	141(64.09)/ 79(35.91)	< 0.0001
**Co-infection, n(%)**				
Influenza A	401(97.33)/ 6(1.46); 8	193(98.97)/ 2(1.03)	208(95.85)/ 4(1.84); 8	0.0799
Influenza B	402(97.57)/ 5(1.21); 8	195(100.00)/ 0(0.00)	207(95.39)/ 5(2.30); 8	0.0101
Tuberculosis	8(1.94)/ 405(98.06); 2	3(1.54)/ 192(98.46)	5(2.29)/ 213(97.71); 2	0.8428
**Symptoms and signs, n(%)**				
heart rate, (per minute)				0.0002
≤ 100	37(15.42)	28(26.17)	9(6.77)	
>100	200(83.33)	77(71.96)	123(92.48)	
Total	240(100.00)	107(100.00)	133(100.00)	
Nasal stuffiness	8(1.94)/ 405(98.06); 2	4(2.05) /191(97.95)	4(1.83) /214(98.17); 2	1.0000
Nasal discharge	12(2.91) /401(97.09); 2	5(2.56)/ 190(97.44)	7(3.21)/ 211(96.79); 2	0.6960
Sneezing	4(0.97) /408(99.03); 3	3(1.54)/ 192(98.46)	1(0.46)/ 216(99.54); 3	0.5414
Sore throat	8(1.94) /405(98.06); 2	4(2.05)/ 191(97.95)	4(1.83)/ 214(98.17); 2	1.0000
Cough	330(79.90) /83(20.10); 2	157(80.51)/ 38(19.49)	173(79.36)/ 45(20.64); 2	0.7700
Sputum production	157(38.01)/ 256(61.99); 2	67(34.36)/ 128(65.64)	90(41.28)/ 128(58.72); 2	0.1478
Chest tightness	264(63.92)/ 149(36.08); 2	113(57.95)/ 82(42.05)	151(69.27)/ 67(30.73); 2	0.0168
Chest pain	12(2.91) /401(97.09); 2	6(3.08)/ 189(96.92)	6(2.75)/ 212(97.25); 2	0.8445
Hemoptysis	7(1.69) /406(98.31); 2	4(2.05)/ 191(97.95)	3(1.38)/ 215(98.62); 2	0.8817
Headache	13(3.15)/ 400(96.85); 2	10(5.13)/ 185(94.87)	3(1.38)/ 215(98.62); 2	0.0292
Myalgia	49(11.86) /364(88.14); 2	29(14.87)/ 166(85.13)	20(9.17)/ 198(90.83); 2	0.0739
Fatigue	165(40.05) /247(59.95); 3	78(40.21)/ 116(59.79)	87(39.91)/ 131(60.09); 2	0.9509
Digestive symptoms	58(14.04)/ 355(85.96); 2	28(14.36)/ 167(85.64)	30(13.76)/ 188(86.24); 2	0.8615
Discomfort of eye	1(0.24)/ 412(99.76); 2	1(0.51)/ 194(99.49)	0(0.00)/ 218(100.00); 2	0.4722
Cyanosis	48(11.59) /366(88.41); 1	10(5.13)/ 185(94.87)	38(17.35)/ 181(82.65); 1	0.0001
Rhonchial	26(6.30) /386(93.46); 3	13(6.67)/ 181(92.82)	13(5.96)/ 205(94.04); 2	1.0000
Moist rales	76(18.40) /337(81.60); 2	33(16.92)/ 162(83.08)	43(19.72)/ 175(80.28); 2	0.4632
SPO_2_(≤93%/>93%)	279(67.23)/ 136(32.77)	96(49.23)/ 99(50.77)	183(83.18)/ 37(16.82)	< 0.0001
Length of hospitalization, d	12.00(7.00,18.00);	15.00 (11.00,20.00)	10.00(4.00,15.00)	< 0.0001
Length of ICU, d	2.00(0.00,9.00);	0.00 (0.00,6.50);	3.00 (1.00,9.00);	< 0.0001
Temperature, °C	38.50(38.00,39.00)	38.50 (38.00,39.00)	38.50 (38.00,39.00)	0.5281
arterial pressure, mmHg	87.00(79.83,92.83); 21	86.00 (78.00,91.33);2	88.00 (82.00,93.67); 19	0.0005
heart rate, per mimute	89.00(81.00,100.00); 2	90.00 (82.00,100.00)	88.50 (80.00,102.00); 2	0.9356
respiratory rate	22.00(20.00,26.00); 1	22.00 (20.00,26.00)	22.00 (20.00,28.00)	0.7331
<24, (per minute)	251(60.48)	122(62.56)	129(58.64)	0.3642
24–30, (per minute)	87(20.96)	40(20.51)	47(21.36)	
≥ 30, (per minute)	77(18.55)	33(16.92)	44(20.00)	
Total	415(100.00)	195(100.00)	220(100.00)	
SOFA	3.00(1.00,4.00); 4	2.00(1.00, 3.00)	3.00(2.00,5.00); 4	< 0.0001
APACHEII	7.00(5.00,10.00); 4	6.00 (4.00,7.00)	9.00 (7.00,13.00); 4	< 0.0001
**Laboratory Tests**				
Lowest leukocyte, ×10^9^/L	5.44(3.83, 8.50)^b^; 10	4.45 (3.43,5.91)	7.57 (4.63,11.60); 10	< 0.0001
Lowest haemoglobin, g/L	109.00(88.00,123.00); 10	110.00 (91.00,124.00)	108.00 (86.00,122.00); 10	0.5267
Lowest patelets, ×10^9^/L	125.50(53.00,188.00);9	152.00 (86.00,217.00)	91.00 (42.00,165.00); 9	< 0.0001
Lowest neutrophils, ×10^9^/L	5.38(3.00,11.36); 14	3.55 (2.55,8.59)	6.99 (4.30,11.63); 14	< 0.0001
Lowest lymphocyte, ×10^9^/L	0.55(0.35,1.06); 11	0.86 (0.49,1.44)	0.44 (0.28,0.64); 11	< 0.0001
Lowest fibrinogen, g/L	4.10(2.70,5.90); 22	4.50 (3.40,6.50);6	3.60 (2.20,5.30); 16	< 0.0001
Lowest prealbumin, g/L	83.00(45.00,117.00); 10	104.00 (67.00,145.00); 1	64.00 (33.00,98.00); 9	< 0.0001
Lowest albumin, g/L	28.00(25.10,31.40); 8	30.10 (27.60,34.40)	26.00 (23.45,28.70); 8	< 0.0001
Peak D-Dimer, mg/L	10.06(1.15,42.82)	1.58 (0.63,14.04)	27.31 (7.60,58.76)	< 0.0001
Peak total bilirubin, μg/L	18.95(13.00,32.30); 13	15.40 (11.70,30.00); 2	22.50 (15.39,32.76); 11	0.0019
Peak alanine aminotransferase, U/L	49.00(31.00,77.00); 12	48.00 (30.00,68.00)	50.50 (31.50,87.00); 12	0.1652
Peak aspartate aminotransferase, U/L	43.00(29.00,72.00); 11	34.00 (24.00,50.00)	57.00 (36.00,108.00); 11	< 0.0001
Peak blood urea nitrogen, mmol/L	9.20(5.97,18.00); 14	6.66 (5.03,9.79); 1	14.90 (8.10,24.30); 13	< 0.0001
Peak serum creatinine, μg/L	82.00(59.40,139.80); 13	70.70 (53.70,89.30)	120.70 (69.30,276.80); 13	< 0.0001
Peak triglycerides, mmol/L	2.15(1.42,3.43); 14	2.16 (1.42,4.53);1	2.10 (1.41,3.27); 13	0.5062
Peak ferritin, μg/L	927.22(492.02,2000.00); 18	600.08 (356.00,987.50);6	1675.09 (837.84,2000.00);12	< 0.0001
Peak Interleukin −6, pg/mL	11.18(7.92,19.57); 26	9.32 (6.90,13.50); 9	13.89 (9.99,27.90); 17	< 0.0001
Peak erythrocyte sedimentation rate, mm/h	60.00(43.00,79.00); 22	61.00 (45.30,88.00); 4	59.35 (40.00,73.00); 18	0.0527
Peak C-reactive protein, mg/L	122.60(30.50,160.00); 12	49.40 (8.70,121.90); 3	160.00 (111.00,160.40); 9	< 0.0001
Peak procalcitonin, ng/mL	0.20(0.06,2.87); 17	0.07 (0.05,0.23); 4	1.08 (0.16,6.40); 13	< 0.0001
Peak lactate dehydrogenase, U/L	429.00(274.00,699.00); 10	300.50 (221.00,403.00); 1	675.00 (464.00,1095.00); 9	< 0.0001
Peak creatine kinase isoenzyme, U/L	27.00(17.00,66.00); 16	19.50 (14.00,51.00); 1	34.00 (20.00,84.00); 15	< 0.0001
Peak Troponin, pg/mL	10.85(3.50,99.00); 13	4.40 (1.70,9.10); 1	70.50 (11.20,823.05); 12	< 0.0001

Abbreviations: *ARDS* Acute Respiratory Distress Syndrome, *DIC* Disseminated Intravascular Coagulation, *COPD* Chronic Obstructive Pulmonary Disease, *ECMO* Extracorporeal Membrane Oxygenation, *CRRT* Continuous Renal Replacement Therapy, *ICU* Intensive Care Unit, *SOFA* Sequential Organ Failure Assessment, *APACHE* Acute Physiology, Age, Chronic Health Evaluation

Data are expressee as: ^a^No. (%) yes/no; no. missing (if applicable); ^b^Median (range); no. missing (if applicable)

*P* value compare between survivor group vs non-survivor group, *P* < 0.05 means had significantly different

### Clinical characteristics and laboratory findings of COVID-19 associated with suspected sHLH according to HScore

Of the 415 patients the available data was only sufficient to generate HScores for 408. Of these 408 patients, 41 (10.05%) had a HScore≥169 and were identified as suspected sHLH. Conversely, 279 (68.38%) had a HScore≤98 were considered negative for suspected sHLH. The remaining 88 patients had HScores 98–169 and were not categorized.

Mortality in the suspected sHLH positive group was significantly higher than that in the suspected sHLH negative group (100.00% vs 34.77%, *P* < 0.001) (Table 2).

**Table 2:** Clinical Characteristics and Laboratory Findings of COVID-19 according to HScore (≤98 as sHLH negative, ≥169 as sHLH positive, 98–169 as uncertain)

	≤98 (*n* = 279)	≥169(*n* = 41)	98–169(*n* = 88)	*P* value^c^
outcome				< 0.0001
Survivor, n(%)	182(65.23)	0(0.00)	13(14.77)	
Non-survivor, n(%)	97(34.77)	41(100.00)	75(85.23)	
Total	279(100.00)	41(100.00)	88(100.00)	
Gender, Male/Female, n(%)	153(54.84)/ 126(45.16)	27(65.85)/14(34.15)	60(68.18)/28(31.82)	0.0537
Age, years, Mean (SD)	63.00(52.00,72.00)	63.00(52.00,71.00)	65.00(57.00,72.00)	0.2419
age, years, n(%)				0.1869
<40	17(6.09)	3(7.32)	3(3.41)	
40–60	101(36.20)	11(26.83)	25(28.41)	
≥ 60	161(57.71)	27(65.85)	60(68.18)	
ARDS, n(%)	150(53.76)/129(46.24)^a^	34(85.00)/6(15.00); 1	68(80.95)/16(19.05); 4	< 0.0001
DIC, n(%)	6(2.17)/ 271(97.83); 2	4(10.53)/34(89.47);3	10(12.66)/69(87.34); 9	0.0004
Liver, n(%)	36(13.00)/241(87.00); 2	12(31.58)/26(68.42); 3	30(37.97)/49(62.03); 9	< 0.0001
Heart, n(%)	50(18.05)/227(81.95); 2	17(44.74)/21(55.26); 3	35(44.30)/44(55.70); 9	< 0.0001
Kidney, n(%)	17(6.14)/260(93.86); 2	16(42.11)/22(57.89); 3	26(32.91)/53(67.09); 9	< 0.0001
Shock, n(%)	31(11.19)/246(88.81); 2	20(52.63)/18(47.37); 3	34(43.04)/45(56.96); 9	< 0.0001
Smoking history, n(%)	14(5.04)/264(94.96); 1	2(4.88)/39(95.12)	4(4.60)/83(95.40); 1	0.9111
Alcohol history, n(%)	16(5.76)/262(94.24); 1	1(2.44)/40(97.56)	4(4.60)/83(95.40); 1	0.3577
**Comobidities**				
Diabetes,n(%), n(%)	50(17.99)/228(82.01); 1	9(21.95)/32(78.05)	20(22.73)/68(77.27)	0.5630
Hypertension, n(%)	97(34.89)/181(65.11); 1	11(26.83)/30(73.17)	38(43.18)/50(56.82)	0.1639
Chronic heart failure, n(%)	29(10.43)/249(89.57); 1	2(4.88)/39(95.12)	7(7.95)/81(92.05)	0.2138
COPD, n(%)	5(1.80)/273(98.20); 1	2(4.88)/39(95.12)	6(6.82)/82(93.18)	0.0537
Stroke, n(%)	18(6.47)/260(93.53); 1	0(0.00)/41(100.00)	8(9.09)/80(90.91)	0.3853
Tumor, n(%)	11(3.96)/267(96.04); 1	2(4.88)/39(95.12)	5(5.68)/83(94.32)	0.5927
Chronic liver disease, n(%)	6(2.16)/272(97.84); 1	2(4.88)/39(95.12)	6(6.82)/82(93.18)	0.0907
Chronic kidey disease, n(%)	7(2.52)/271(97.48); 1	2(4.88)/39(95.12)	2(2.27)/86(97.73)	0.5200
Hepatitis B,n(%)	11(3.94)/268(96.06)	3(7.32)/38(92.68)	1(1.15)/86(98.85); 1	0.7610
HIV, n(%)	0(0.00)/279(100.00)	0(0.00)/41(100.00)	0(0.00)/87(100.00); 1	1.0000
Autoimmue disease, n(%)	7(2.52)/271(97.48); 1	3(7.32)/38(92.68)	6(6.82)/82(93.18)	0.0422
**Respiratory support, n(%)**				< 0.0001
Intranasal oxygen inhalation	129(47.43)	3(8.11)	13(16.46)	
Mask oxygen Inhalation	16(5.88)	1(2.70)	1(1.27)	
High Flow	48(17.65)	3(8.11)	14(17.72)	
Non-invasive ventilation	35(12.87)	12(32.43)	26(32.91)	
Invasive ventilaiton	17(6.25)	14(37.84)	22(27.85)	
ECMO	1(0.37)	3(8.11)	0(0.00)	
**CRRT, n(%)**	50(17.99)/228(82.01); 1	9(21.95)/32(78.05)	20(22.73)/68(77.27)	0.5630
**Inotropic support, n(%)**	33(11.83)/ 246(88.17)	21(51.22)/ 20(48.78)	30(34.09)/ 58(65.91)	< 0.0001
**Corticosteroid treatment, n(%)**	133(47.67)/ 146(52.33)	35(85.37)/ 6(14.63)	55(62.50)/ 33(37.50)	< 0.0001
**Co-infection, n(%)**				
Influenza A	3(1.08)/ 273(98.56); 3	1(2.44)/ 38(92.68); 2	2(2.27) /84(95.45); 2	0.0924
Influenza B	1(0.36)/275(99.28); 3	1(2.44)/ 38(92.68); 2	3(3.41) /83(94.32); 2	0.0127
Tuberculosis	4(1.44) /274(98.56);1	1(2.44)/ 40(97.56)	3(3.41)/ 85(96.59)	1.0000
**Symptoms and signs, n(%)**				
heart rate, (per minute)				0.6759
≤ 100	32(21.77)	2(3.57)	4(6.67)	
>100	113(76.87)	26(92.86)	56(93.33)	
Total	147(100.00)	28(100.00)	60(100.00)	
Nasal stuffiness	5(1.80) /273(98.20); 1	2(4.88) /39(95.12)	1(1.14) 87(98.86)	1.0000
Nasal discharge	6(2.16)/272(97.84); 1	5(12.20)/ 36(87.80)	1(1.14)/87(98.86)	0.0086
Sneezing	3(1.08)/ 275(98.92); 1	0(0.00)/40(100.00); 1	1(1.14)/87(98.86)	1.0000
Sore throat	4(1.44)/274(98.56); 1	1(2.44)/40(97.56)	3(3.41)/ 85(96.59)	1.0000
Cough	226(81.29)/ 52(18.71); 1	30(73.17)/11(26.83)	69(78.41)/ 19(21.59)	0.4468
Sputum production	104(37.41)/174(62.59); 1	16(39.02)/25(60.98)	33(37.50)/55(62.50)	0.9802
Chest tightness	172(61.87)/106(38.13); 1	30(73.17) /11(26.83)	58(65.91) /30(34.09)	0.3367
Chest pain	7(2.52) /271(97.48)	1(2.44) /40(97.56)	4(4.55)/ 84(95.45)	0.6647
Hemoptysis	5(1.80) 273(98.20); 1	2(4.88) /39(95.12)	0(0.00) /88(100.00)	1.0000
Headache	9(3.24)/ 269(96.76); 1	2(4.88) /39(95.12)	2(2.27)/ 86(97.73)	0.8098
Myalgia	34(12.23) /244(87.77); 1	3(7.32) /38(92.68)	12(13.64) /76(86.36)	0.5736
Acratia	102(36.82)/175(63.18);2	26(63.41) /15(36.59)	35(39.77)/ 53(60.23)	0.0052
Digestive symptoms	36(12.95) /242(87.05)	9(21.95)/ 32(78.05)	13(14.77)/ 75(85.23)	0.3021
Discomfort of eye	1(0.36)/ 277(99.64); 1	0(0.00)/ 41(100.00)	0(0.00)/ 88(100.00)	1.0000
Cyanosis	27(9.71) /251(90.29); 1	10(24.39) /31(75.61)	7(7.95)/ 81(92.05)	0.0393
Rhonchial	18(6.47) 259(93.17); 1	4(9.76) /37(90.24)	4(4.55)/ 84(95.45)	1.0000
Moist rales	51(18.35)/ 227(81.65)	12(29.27) /29(70.73)	12(13.64) 76(86.36)	0.1028
SPO_2_(≤93%/>93%)	173(62.01)/ 106(37.99)	32(78.05)/ 9(21.95)	67(76.14)/ 21(23.86)	0.0131
Length of hospitalization, d	12.00(8.00,18.00)^b^	17.00(11.00,22.50)	12.00(7.00,16.00)	0.0029
Length of ICU, d	0.00(0.00,6.00)	9.50(3.50,12.00)	3.00(0.50,12.00)	< 0.0001
Temperature, °C	38.50(38.00,39.00)	38.50(38.00,38.90)	38.90(38.00,39.00)	0.4406
Arterial pressure, mmHg	87.00(78.33,93.00)	86.58(81.50,91.33)	86.75(81.33,93.50)	0.1593
Heart rate, per mimute	89.00(82.00,99.00); 1	90.0081.00,100.00	88.00(80.00,102.00)	0.5895
Respiratory rate				0.5070
<24, (per minute)	164(58.78)	26(63.41)	58(65.91)	
24–30, (per minute)	65(23.30)	5(12.20)	15(17.05)	
≥ 30, (per minute)	50(17.92)	10(24.39)	15(17.05)	
Total	279(100.00)	41(100.00)	88(100.00)	
SOFA	2.00(1.00,3.00)	3.00(1.00,4.00)	3.00(2.00,5.00)	< 0.0001
APACHEII	7.00(5.00,9.00)	8.00(6.00,11.00)	8.00(6.00,13.00)	0.0010
**Laboratory Tests**				
Lowest leukocyte, ×10^9^/L	5.08(3.74,7.83)^b^; 4	6.97(4.38,9.31)	6.77(3.81,10.53); 1	0.0118
Lowest haemoglobin, g/L	112.00(94.00,124.00); 4	95.00(77.00,106.00)	106.00(78.00,122.00); 1	0.0020
Lowest patelets, ×10^9^/L	151.00(81.00,197.00); 3	61.00(27.00,101.00)	83.00(26.00,144.00); 1	< 0.0001
Lowest neutrophils, ×10^9^/L	4.73(2.76,11.46); 8	5.10(3.32,7.39)	7.02(3.97,12.72); 1	0.0804
Lowest lymphocyte, ×10^9^/L	0.69(0.44,1.22); 5	0.33(0.18,0.53)	0.43(0.28,0.66); 1	< 0.0001
Lowest fibrinogen, g/L	4.50(3.24,6.35); 15	2.40(1.60,3.46)	3.80(2.10,5.40); 2	< 0.0001
Lowest prealbumin, g/L	93.00(59.00,127.00); 4	51.00(28.00,85.00)	55.00(29.00,89.00); 1	< 0.0001
Lowest albumin, g/L	28.90(26.20,32.30); 2	24.40(22.30,27.60)	26.60(23.40,28.90); 1	< 0.0001
Peak D-Dimer, mg/L	4.58(0.80,32.09); 5	47.82(20.06,80.00)	17.83(3.46,63.70); 1	< 0.0001
Peak total bilirubin, μg/L	16.60(12.20,30.00); 6	23.98(17.38,37.80)	25.20(16.60,32.30); 1	0.0007
Peak alanine aminotransferase, U/L	47.00(28.00,67.00); 6	57.00(36.00,122.00)	62.00(38.00,109.00); 1	0.0001
Peak aspartate aminotransferase, U/L	38.00(26.00,55.00); 5	85.00(52.00,215.00)	65.00(40.00,121.00); 1	< 0.0001
Peak blood urea nitrogen, mmol/L	7.50(5.30,11.00); 8	21.66(13.50,35.30)	14.90(8.50,24.00); 1	< 0.0001
Peak serum creatinine, μg/L	72.95(55.60,99.00); 7	246.10(146.00,413.70)	105.20(67.40,263.10); 1	< 0.0001
Peak triglycerides, mmol/L	1.95(1.31,3.27); 8	3.27(2.33,4.27)	2.20(1.58,3.43); 1	0.0001
Peak ferritin, μg/L	710.11(433.23,1276.37); 11	2000.00(2000.00,2000.00)	2000.00(962.51,2000.00); 2	< 0.0001
Peak Interleukin −6, pg/mL	10.20(7.26,14.98); 16	23.41(11.73,60.57); 1	14.60(8.56,26.90); 4	< 0.0001
Peak erythrocyte sedimentation rate, mm/h	60.00(43.00,80.00); 10	65.50(49.20,79.00); 3	58.30(39.00,74.00); 4	0.5133
Peak C-reactive protein, mg/L	76.68(15.60,160.00); 5	160.00(160.00,206.40)	160.00(121.20,198.80); 2	< 0.0001
Peak procalcitonin, ng/mL	0.11(0.05,0.50); 10	4.21(2.63,15.22)	0.98(0.19,4.90); 2	< 0.0001
Peak lactate dehydrogenase, U/L	349.00(241.00,534.00); 4	1096.00(708.00,1676.00)	662.00(432.00,944.00); 1	< 0.0001
Peak creatine kinase isoenzyme, U/L	21.50(15.00,52.50); 9	47.00(32.00,85.00)	38.00(21.00,77.00); 1	< 0.0001
Peak Troponin, pg/mL	6.15(2.40,24.60); 7	303.10(33.80,2202.10)	51.50(6.44,591.40); 1	< 0.0001

Abbreviations: *ARDS* Acute Respiratory Distress Syndrome, *DIC* Disseminated Intravascular Coagulation, *COPD* Chronic Obstructive Pulmonary Disease, *ECMO* Extracorporeal Membrane Oxygenation, *CRRT* Continuous Renal Replacement Therapy, *ICU* Intensive Care Unit, *SOFA* Sequential Organ Failure Assessment, *APACHE* Acute Physiology, Age, Chronic Health Evaluation

Data are expressee as: ^a^No. (%) yes/no; no. missing (if applicable); ^b^Median (range); no. missing (if applicable)

^c^ *P* value compare between Hscore ≥169 group vs Hscore ≤98 group, *P* < 0.05 means had significantly different

There were no statistically significant differences in age and gender between suspected sHLH negative and positive groups. Clinical symptoms and signs were similar in suspected sHLH positive group and negative group, except cyanosis was more frequent in suspected sHLH positive patients (*P* = 0.03). For comobidities, suspected sHLH positive patients had significantly higher autoimmue disease (*P* = 0.042).

Compared to the suspected sHLH negative group, the suspected sHLH positive group had significantly higher prevalence of complications including ARDS, DIC, liver injury, heart injury, AKI and, shock as well as high SOFA scores (all *P* < 0.05) (Table 2).

Several laboratory findings were also statistically different between patients with suspected sHLH compared to those without, such as lower haemoglobin (Hb), platelets, lymphocytes, fibrinogen, pre-albumin, albumin, and higher D-Dimer, total bilirubin (Tbil), alanine aminotransferase (ALT), AST, triglycerides (TG), BUN, sCr, IL-6, PCT, LDH, ferritin, CK-MB, troponin,

With regard to treatments, of 41 suspected sHLH suspected patients, 8.11% (*n* = 3) received high-flow nasal cannula, 32.43% (*n* = 12) received non-invasive positive-pressure ventilation (NPPV), 37.84% (*n* = 14) were intubated, 8.11% (*n* = 3) received extracorporeal membrane oxygenation, 21.95% (*n* = 9) received continuous renal replacement therapy (CRRT), 51.22% (*n* = 21) received inotropic support and 85.37% (*n* = 35) received corticosteroid treatment. Except CRRT (*P* = 0.56), all above-mentioned treatments in suspected sHLH positive group were significantly more common compared to suspected sHLH negative group (all *P* < 0.05) (Table 2).

### Risk factors for mortality of severe COVID-19

High SOFA score, low leukocyte, low lymphocyte, low prealbumin, high AST, high CRP, high LDH and old age, were all significantly associated with death in severe COVID-19 patients by logistic regression analysis (all *P* < 0.05) (Table 3). In addition a higher HScore was also significantly associated with death in severe COVID-19 patients. This association was significant in comparison of patients with HScore ≤98 versus patients with HScore ≥169 (Odds Ratio = 22.77, 95% Confidence Interval, 2.23–232.80, *P* = 0.008), and was also significant with comparison of patients with HScore ≤98 versus patients with HScore 98–169 (Odds Ratio = 4.02, 95% Confidence Interval, 1.55–10.44, *P* = 0.004).

**Table 3:** Logistic regression analyses of potential risk factors of Hemophagocytic lymphohistiosytosis (HLH)

	Regression Coefficient	OR (Odds Ratio)	95% CI	*P* value
SOFA	0.221	1.247	1.010–1.539	0.040
Lowest haemoglobin, g/L	0.003	1.003	0.983–1.023	0.769
Lowest platelet,×10^9^/L	0.008	1.008	1.000–1.015	0.038
Lowest neutrophils, ×10^9^/L	0.025	1.026	0.987–1.066	0.195
Lowest fibrinogen, g/L	0.042	1.043	0.999–1.089	0.052
Lowest prealbumin, g/L	0.006	1.006	0.994–1.017	0.332
Peak D-Dimer, mg/L	−0.004	0.996	0.993–1.00	0.061
Peak aspartate aminotransferase, U/L	−0.000	1.000	0.999–1.001	0.910
Peak serum creatinine, μg/L	−0.002	0.998	0.996–1.000	0.126
Peak triglycerides, mmol/L	−0.300	0.741	0.611–0.900	0.003
Peak ferritin, ng/mL	−0.002	0.998	0.997–0.999	< 0.001
Peak Interleukin −6, pg/mL	−0.000	1.000	0.999–1.000	0.175
Peak C-reactive protein, mg/L	−0.002	0.998	0.990–1.005	0.510
Peak lactate dehydrogenase, U/L	−0.000	1.000	0.999–1.000	0.639

Abbreviations: *SOFA* Sequential Organ Failure Assessment

*P* value compare between Hscore ≥169 group vs Hscore <169 group, *P* < 0.05 means had significantly different

### Risk factors related to COVID-19 associated suspected sHLH

Multivariable logistic regression analyses found that independent risk factors for discriminating suspected sHLH in COVID-19 included high triglycerides (Odds Ratio, 0.74; 9% Confidence Interval, 0.61–0.90), high ferritin (Odds Ratio, 0.99; 95% Confidence Interval, 0.997–0.999), low platelets (Odds Ratio, 1.0079; 95% Confidence Interval, 1.00–1.01) (Table 4).

**Table 4:** Logistic regression analyses of potential risk factors of mortality in severe COVID-19 patients

	Regression Coefficient	OR (Odds Ratio)	95% CI	*P* value
SOFA	0.317	1.373	1.006–1.712	0.005
Lowest leukocyte, ×10^9^/L	0.277	1.319	1.174–1.491	< 0.001
Lowest neutrophils, × 10^9^/L	−0.011	0.989	0.976–1.001	0.080
Lowest lymphocyte, ×10^9^/L	0.316	1.372	1.089–1.729	0.007
Lowest prealbumin, g/L	−0.012	0.987	0.979–0.994	0.001
Lowest albumin, g/L	0.012	1.013	0.988–1.038	0.300
Peak D-Dimer, mg/L	−0.001	0.998	0.991–1.005	0.632
Peak aspartate aminotransferase, U/L	−0.004	0.995	0.992–0.997	0.000
Peak blood urea nitrogen, mmol/L	0.0004	1.000	0.989–1.011	0.931
Peak serum creatinine, μg/L	0.001	1.001	0.999–1.003	0.131
Peak Interleukin −6, pg/mL	−0.000	0.999	0.998–1.000	0.880
Peak C-reactive protein, mg/L	0.006	1.006	1.000–1.012	0.036
Peak lactate dehydrogenase, U/L	0.003	1.003	1.001–1.005	< 0.001
Peak creatine kinase isoenzyme, U/L	0.003	1.003	0.999–1.008	0.080
Peak Troponin, pg/mL	0.000	0.999	0.999–1.000	0.293
Age, y	0.057	1.059	1.030–1.088	< 0.001
Hscore 98–169 vs ≤98	1.393	4.028	1.553–10.444	0.004
Hscore ≥169 vs ≤98	3.125	22.770	2.227–232.800	0.008

Abbreviations: *SOFA* Sequential Organ Failure Assessment

*P* value compare between survivor group vs nonsurvivor group, *P* < 0.05 means had significantly different

**Fig. 1:**
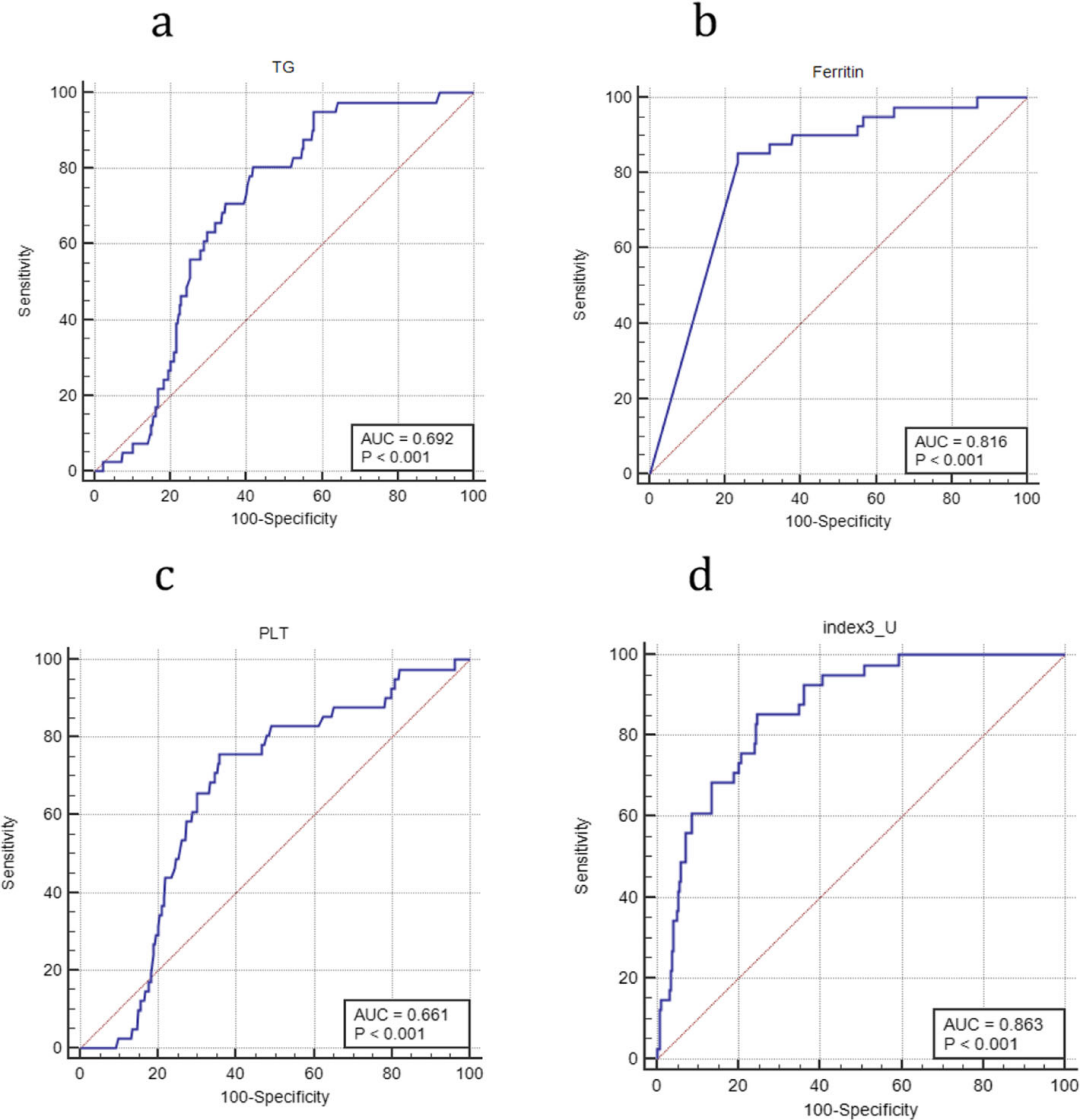
Receiver operating characteristic curve for peak TG (**a**), ferritin (**b**), PLT (**c**) respectively. Receiver operating characteristic curve for the multivariable logistic regression model, including TG, ferritin, PLT (**d**). Abbreviations: AUC, area under the curve; TG, triglyceride; PLT, platelet

Receiver operating characteristic curve analysis indicated that high ferritin may alone diagnose suspected sHLH in COVID-19, which had an area under the curve (AUC) of 0.816 (*P* < 0.01, sensitivity 85.4%, specificity 76.6%, cut-off value > 1922.58 ng/mL). A combination of high ferritin (cut-off value > 1922.58 ng/mL), high triglycerides (cut-off value > 2.28 mmol/L), and low platelets (cut-off value < 101 × 10^9^/L) had an AUC of 0.863 (*P* < 0.001, sensitivity 85.4%, specificity 75.4%) (Fig. 1).

**Fig. 2:**
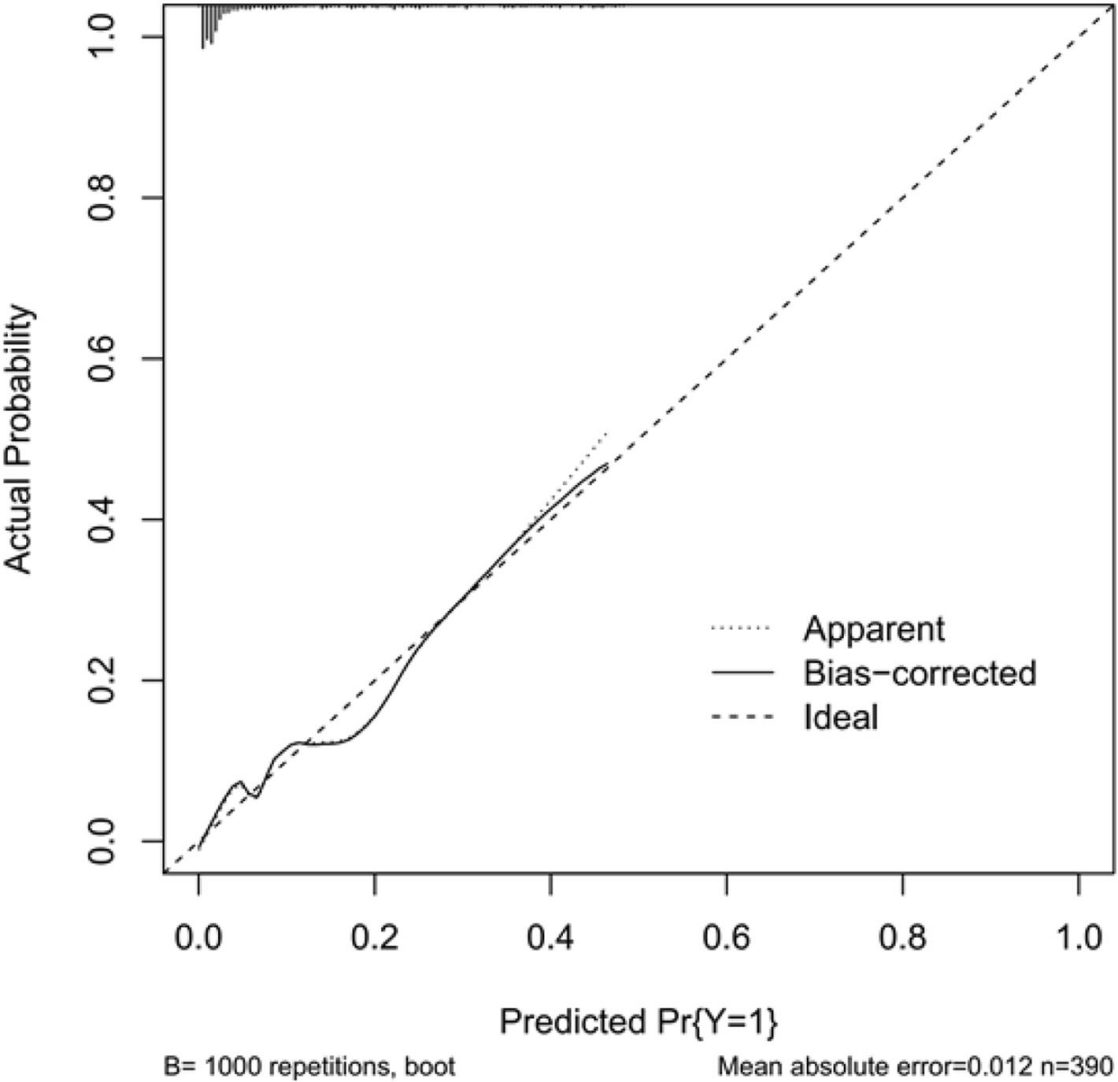
Internal validation to validate a prediction model

Internal validation was conducted using a bootstrapping strategy (1000 repetitions), the results achieved a C-index of 0.863 (95%CI 0.838–0.889). The calibration plot was shown Fig. 2.

## Discussion

Herein, we present data that demonstrates the prognostic value of HScores, and three independent risk factors for high HScores in a specific cohort of severe COVID-19 patients. In this cohort, mortality was 53.01% in patients with severe COVID-19 and 100% in patients with suspected sHLH. Furthermore, we observed a significantly higher incidence of multi-organ dysfunction in patients with suspected sHLH. The frequency rate of suspected sHLH in severe COVID-19 patients was 10.05% (41 of 408). We assessed the clinical factors associated with suspected sHLH including hemoglobin, platelets, lymphocytes, fibrinogen, pre-albumin, albumin, D-dimer, total bilirubin, blood urea nitrogen, serum creatinine, triglycerides, ferritin, interleukin-6, C-reactive protein, pro-calcitonin, lactate dehydrogenase, creatinine kinase-MB and troponin. The independent risk factors of suspected sHLH in this study were low platelet count(< 101 × 10^9^/ml), high triglycerides(>2.28 mmol/L) and high ferritin (>1922.58 ng/mL).

There are two major kinds of HLH: primary and secondary. Primary HLH is related to gene defects – wherein typically CD8 and Natural Killer (NK) mediated cytotoxic genes are defective. Secondary HLH is acquired through infection, malignant conditions, and autoimmune diseases. It is a rare life-threatening complication characterized by uncontrolled activation of CD8 + T cells and NK cells, cytokine storm, and severe organ dysfunction [6, 7]. However, suspected sHLH lacks specific clinical characters [7]. Our study found that severe COVID-19 patients with suspected sHLH had similar clinical manifestations including body temperature, heart rhythm, respiration, and clinical symptoms as those without suspected sHLH. The overlap of clinical features impedes the diagnosis of suspected sHLH in severe infectious diseases especially in the case of cytopenias, prolonged fevers, and non-responsiveness to treatment [6, 7]. According to recommendations for the management of hemophagocytic lymphohistiocytosis in adults, reevaluation of the clinical condition should be frequent (at least every 12 h) to determine if additional HLH-directed therapy is needed [16]. Therefore, the assessment of HScore in severe COVID-19 patients could improve the detection rates of suspected sHLH and promote early targeted-treatment. The HLH-2004 protocol was also widely used for the diagnosis of hemophagocytic lymphohistiocytosis. We did not apply HLH-2004 as diagnosis tool because most of the patients in our study did not have data on four diagnostic items of HLH-2004 protocol, including haemophagocytosis in bone marrow or spleen or lymph nodes, low or absent natural killer cell activity, soluble CD25 (soluble interleukin-2 receptor).

Progression of COVID-19 into organ failure results from a catastrophic overreaction of the immune system to the virus, known as a “cytokine storm”. Cytokine storms are characterized by high levels of IL-6, C-reactive protein and ferritin and causes immune cell-mediated tissue damage and catastrophic organ failure [6, 7]. As demonstrated in this study, the levels of IL-6, C-reactive protein, and ferritin were significantly higher in the suspected sHLH positive group than in the suspected sHLH negative group. It has been previously suggested that the presence of high ferritin syndrome in COVID-19 was a high-risk factor for suspected sHLH [3, 5]. Consistent with this, we observed that increased peak ferritin was associated with suspected sHLH and with increased fatality in severe COVID-19 (*P* < 0.001). It is important to consider that a higher ferritin level than 1922 ng/ml may be used as a screening indicator for suspected sHLH related to COVID-19.

Hypertriglyceridemia (fasting, equal to, or greater than 1.5 mmol/L) is one of the current diagnostic criteria for suspected sHLH [15]. Our results showed that hypertriglyceridemia (> 2.28 mmol/L) might be an independent risk factor to determine severe COVID-19 associated suspected sHLH. It also is easily available and standardized as laboratory diagnostic testing in clinical practice. The hyperinflammatory response contributes to hypertriglyceridemia via prolonged and intense T-lymphocyte and macrophage signaling. The proinflammatory signaling induces elevated IL-6 which mobilizes free fatty acids from adipocytes, decreased lipoprotein lipase activity and increased tumor necrosis factor alpha expression [17]. Furthermore, metabolic syndrome, wherein hypertriglyceridemia is a hallmark, also has characteristic increased serum ferritin levels, suggesting similar pathogenic mechanisms [18].

Thrombocytopenia is reported to be common in COVID-19 patients and is considered a risk factor for increased disease severity and mortality [19]. In our cohort study, severe COVID-19 patients had a low median platelet count (125.50 × 10^9^/L). The mechanism underlying the low count of platelet in COVID-19 is multi-factorial and complex. First, SARS-CoV-2 damages endothelial cells triggering platelet activation and thrombosis in multi-organ, causing vast platelet consumption [19, 20]. Second, excessive inflammation in COVID-19 may induce venous and arterial thromboembolism. The incidence of thrombus in ICU patients with COVID-19 was found to be as high as 31% [21]. Moreover, suspected sHLH leads to depletion of blood cells and can cause cytopenia such as thrombocytopenia [6]. Therefore, we expected that platelets would be much lower in suspected sHLH patients. In the present study, we observed that platelet count, a simple and readily available biomarker, lower than 101 × 10^9^/L is an independent risk factor and can be used to differentiate patients with suspected sHLH in this severe COVID-19 cohort. Thus, high ferritin, hypertriglyceridemia and low platelets are predictive of sHLH and should be measured in patients with severe COVID-19, to identify and facilitate specific treatment of sHLH. In addition, the prediction value of “peak TG, ferritin, and low PLT” in COVID-19 patients were supported by a series of previous studies [22–24].

We found that 41 patients who fulfilled suspected sHLH probability diagnosis according to HScore succumbed to COVID-19, while suspected sHLH negative patients had a survival rate of 68.38%. One of the possible reasons for the high mortality is the lack of targeted-treatment for suspected sHLH. Adult HLH patients with dyspnea, diarrhea, diffuse bleeding, jaundice and septicemia accompanied by organ failure were characterized by extreme symptoms of severe suspected sHLH, which has very poor prognosis [25]. These signs and symptoms, along with significant disease progression, provide a risk signature for possible HLH, consistent with the clinical manifestations of COVID-19 with cytokine storm [26]. Recently research indicated that suspected sHLH related to COVID-19 received IL-6 antagonistic therapy and Anakinra (recombinant soluble receptor antagonist of IL-1β and IL-1α) [27, 28]. Several drugs targeting specific cytokines are in on-going clinical trials in patients with COVID-19 [29] and the earliest possible detection of sHLH is of the utmost importance.

Our study has several limitations. First, in the severe COVID-19 group, some cases of suspected sHLH may have been missed, suggesting that suspected sHLH may have been underestimated in this study. The lack of definite indicators in the 88 patients with HScore ranging between 98 and 169 could have led to the underestimation of suspected sHLH. Second, all patients lacked specific diagnostic indicators for sHLH, such as laboratory test assessing soluble CD25 and NK-cell activity due to insufficient medical resources to provide these tests during the COVID-19 outbreak. As a retrospective study, it is difficult to infer a definite causality between sHLH and severe COVID-19. Therefore, we selected HScores ≥169 as the positive cut-off value and HScores ≤98 as the negative cut-off value to prevent overestimating suspected sHLH in these patients. Third, the upper limit of detection for ferritin was 2000 ng/ml, which might reduce the specificity of ferritin as a predictor of suspected sHLH. Fourth, the clinical parameters of the liver/spleen and cytological results of bone marrow aspiration were virtually absent in all patients. Fifth, there is no second validation cohort for the prediction model. In order to overcome the question, internal validation was conducted using bootstrapping (1000 repetitions), the results achieved a C-index 0.863(95%CI 0.838–0.889).

## Conclusion

Although HScore has been widely used in various infectious diseases to identify sHLH, the criteria were not specifically studied for use in COVID-19 patients. It is important to find the clinical markers that predict mortality in COVID-19 patients. We recommend HScore for early suspected sHLH screening in severe COVID-19 patients with high ferritin, high triglyceride and low platelet levels. Prospective studies are needed to inform whether it is possible to utilize these markers to determine suspected sHLH diagnosis to identify at-risk populations to target for HLH therapy in severe COVID-19 in the future.

## Data Availability

The data that support the findings of this study are available from the corresponding author on reasonable request. Participant data without names and identifiers will be made available after approval from the corresponding author. After publication of study findings, the data will be available for others to request. The research team will provide an email address for communication once the data are approved to be shared with others.

## Abbreviations

sHLH: Secondary hemophagocytic lymphohistiocytosis
SARS-CoV-2: Severe acute respiratory syndrome coronavirus 2
COVID-19: Corona virus disease 2019
HScore: Hemophagocytosis score
EBV: Epstein-Barr Virus
CMV: Cytomegalovirus
HIV: Human immunodeficiency virus
SOFA: Sequential Organ Failure Assessment
ARDS: Acute respiratory distress syndrome
AKI: Acute kidney injury; Fib, fibrin/fibrinogen
DIC: Disseminated intravascular coagulation
SGOT: Serum glutamic oxaloacetic transaminase
ASAT: Recombinant aspartate aminotransferase
APACHE II: Acute Physiology and Chronic Health Evaluation II
AST: Aspartate aminotransferase
BUN: Blood urea nitrogen
sCr: Serum creatinine
IL-6: Interleukin − 6
CRP: C-reactive protein
PCT: Procalcitonin
LDH: Lactate dehydrogenase
CK-MB: Creatine kinase isoenzyme
Hb: Haemoglobin; Tbil, total bilirubin
ALT: Alanine aminotransferase
TG: Triglycerides
NPPV: Non-invasive positive-pressure ventilation
CRRT: Continuous renal replacement therapy
AUC: Area under the curve
C-index: Concordance index
NK: Natural Killer
